# Patients’ views on the use of artificial intelligence in healthcare: Artificial Intelligence Survey Aachen (AISA)—a prospective survey

**DOI:** 10.1186/s13244-025-02159-3

**Published:** 2026-01-05

**Authors:** Sophie G. Baldus, Martin Wiesmann, Ute Habel, Anna Gerhards, Dimah Hasan, Charlotte S. Weyland, Daniel Truhn, Marian M. Hasl, Benjamin Clemens, Omid Nikoubashman

**Affiliations:** 1 Department of Diagnostic and Interventional Neuroradiology, University Hospital, RWTH Aachen University, Aachen, Germany; 2 Department of Psychiatry, Psychotherapy and Psychosomatics, University Hospital, RWTH Aachen University, Aachen, Germany; 3 Department of Diagnostic and Interventional Radiology, University Hospital, RWTH Aachen University, Aachen, Germany; 4 Projektträger Jülich, Forschungszentrum Jülich GmbH, Jülich, Germany; 5 Department of Diagnostic and Interventional Neuroradiology, University Hospital OWL, University Bielefeld, Bielefeld, Germany

**Keywords:** Artificial intelligence, Machine learning, Patient, Survey, Questionnaire

## Abstract

**Objectives:**

The use of AI is gaining relevance in healthcare. There is limited information regarding the views of patients on AI in healthcare. The aim of our study was to assess the views of patients on the use of AI in healthcare with an on-site questionnaire.

**Materials and methods:**

Patients in our tertiary hospital with a diagnostic imaging appointment were invited to complete a paper-based questionnaire between December 2022 and October 2023. We asked about socio-demographic data, experience, knowledge, and their opinion on the use of AI in healthcare, focusing on the fields (1) diagnostics, (2) therapy, and (3) triage.

**Results:**

Out of a total of 198 patients (mean age 49.41 ± 17.6 years, 99 female), 91.5% stated that they expected benefits from the implementation of AI in healthcare, although 73.4% rated their knowledge of AI as moderate to none. The majority of patients were in favour of using AI in diagnostics (87.2%) and therapy (73.1%), while only 28.2% approved its use in patient triage. 84.0% wanted to be informed about the use of AI in at least one of the mentioned areas. Participants with higher education, higher self-assessed knowledge of AI and personal experience with AI showed greater approval for AI in healthcare.

**Conclusion:**

Our interviewed patients have a rather open attitude towards AI in healthcare, with differentiated views depending on the topic; patients are in favour of the use of AI, especially in diagnostics and to a lesser extent also for therapy support, but they reject its use for triage.

**Critical relevance statement:**

Overall, the results emphasise the need for widespread efforts to address patient concerns about AI in healthcare, including enhancing understanding and acceptance while protecting marginalised groups. This will help clinical radiology to adopt AI more effectively.

**Key Points:**

- There is limited information on patients’ views of AI in healthcare, often focused on specific groups, limiting generalizability.
- Patients are open to AI in healthcare, supporting its use in diagnostics and therapy, but rejecting its use for triage.
- Overall, patients want to be informed about AI usage and participants with higher education and AI experience showed more approval.

**Graphical Abstract:**

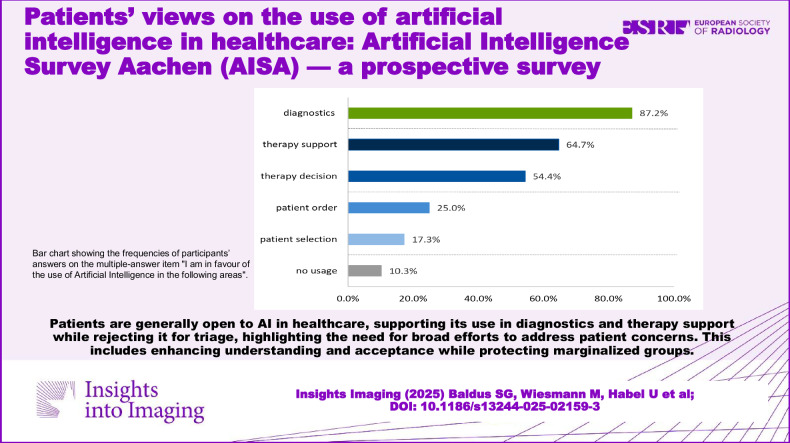

## Introduction

The topic of Artificial Intelligence (AI) permeates many areas of modern life, including healthcare. Today, there are various possible medical AI applications, ranging from radiological assistant systems for imaging interpretation [1], diagnostic tools in dermatology [2], and ophthalmology [3], to algorithms that are supposed to improve detection, diagnosis, classification, and treatment of psychiatric disorders [4, 5].

While many conceptualise AI as a beacon of hope for the future of healthcare, others have expressed concerns with respect to the fast spread of AI models into clinical routine. One important issue is the discrepancy between fast advances in the field of technical aspects of AI on the one hand and patients’ acceptance of AI on the other. Patients’ opinions, concerns and needs are crucial, as the integration of AI into medical practice directly impacts patients’ personal lives and their well-being: Even the best technology is useless when those involved, i.e. caretakers and patients, do not accept it.

Many results of previous studies are not transferable to the general patient population in hospitals: current studies dealing with patients’ views focus on specific use cases such as the diagnosis of skin cancer, radiological exams, or support during neurosurgical procedures, hence limiting the generalizability of their results [6–9]. Also, current studies tend to represent only specific demographic groups, such as women [10, 11], or experienced AI users [12, 13]. In addition, many studies are conducted via online surveys, therefore reaching primarily computer-proficient and rather young individuals [8, 14, 15].

To address some of these issues, the aim of our study was to assess the views of a diverse and representative range of patients on the use of AI in healthcare. We deliberately chose a short on-site questionnaire to approach a broad range of patients, including young and old patients with different sociodemographic backgrounds. We did not examine the acceptance regarding a specific disease or medical speciality but investigated the general opinion regarding AI in healthcare, focusing on the fields of (1) diagnostics, (2) therapy, and (3) triage.

## Materials and methods

After approval from our local ethics committee (number: EK 22-331) and informed consent of our patients, we conducted our survey from December 2022 to October 2023 at the University Hospital RWTH Aachen. The questionnaire was distributed to patients who were scheduled for neuroradiological MR exams, who were older than 18 years and willing to provide informed written consent. We excluded patients receiving acute treatment that could be delayed by participation in the study. Participation was voluntary and pseudonymized after written consent. A total of 200 patients were handed the questionnaire, 198 of whom completed it in full. Overall, 37 participants completed the preliminary questionnaire, and 161 participants completed the updated questionnaire.

The purpose of the current prospective study was to develop a simple and concise questionnaire that was easy to understand and that would allow us to obtain a general picture of patients’ views on the use of AI. We used plain language and aimed for a completion time of less than 10 min. We provided information on the purpose and expected time to complete the questionnaire to the participants and presented the questionnaire in German. In addition to sociodemographic questions about age, gender, highest level of education, migration background, employment status, and current diagnosis, nine items (seven single-choice questions and two multiple-choice questions) dealt with the topic of “Artificial Intelligence in Healthcare” to capture our patients’ subjective knowledge, opinions, and preferences on this subject. Two experienced psychologists and an experienced clinician reviewed the questionnaire that was partially based on items from previous studies on this topic: item 2 [16], item 3 [17], item 5 [8], and item 6 [9] were modified from previous questionnaires. We completed a preliminary test run with an item analysis after 37 participants, according to Perneger et al [18]. Subsequently, we modified our questionnaire by removing the response option “I have no expectation” from item 8, omitting two items altogether due to redundancy and low selectivity, and adding two additional items (numbers 9, 10). The latter items specifically assess the view on the use of AI in specific medical areas, namely: (1) diagnostics, (2) therapy (therapy support and therapy decision making), and (3) triage (patient selection and patient order). The final questionnaire comprised a total of seventeen items and is provided in the Supplementary Material in German and English.

### Statistical analysis

We used descriptive statistics to characterise the sample in terms of age, gender, highest level of education, type of current employment status, and type of disease or reason for appointment. Nominal data are indicated as absolute and relative frequencies ± margin of error (MOE) when indicating questionnaire items; ordinal and non-parametric data are indicated as median with interquartile range (IQR); parametric data are indicated as mean ± standard deviation (SD). Correlations were calculated using Spearman’s correlation coefficient. Chi-Square tests and Fisher’s exact tests were used for dichotomised analyses, depending on sample size. Ordinal and metric data were compared using Mann–Whitney *U*-tests. To identify factors that have an impact on approval of AI, we conducted a binary logistic regression test (enter method, indicating the odds ratio (OR) and 95% confidence intervals (CI)), including age, gender, migration background, education, employment status, knowledge of AI, personal experience with AI, general acceptance of AI, and expectations from AI. For statistical analyses, item 4 (acceptance of AI) was dichotomised into acceptance vs neutral and negative attitudes. *p*-values with an alpha level of < 0.05 were defined as significant. Data were analysed with IBM SPSS Statistics (Version 29, IBM, Armonk, NY).

## Results

### Demographics and questionnaire results

We included the answers of all 198 patients in our analysis. Sociodemographic characteristics can be found in Table 1. In summary, the median age of respondents was 52.5 years [IQR, 34.75–61.25], ranging from 18 to 87 years. Germany was the most common country of birth (91.2%). Compared to the population in our federal state [19], people with a migration background (11.2% vs 28.7%) and with lower school degrees (13.4% vs 27.2%) were underrepresented in our study population [19, 20].

**Table 1:** Sociodemographic characteristics of the study population

Variable	Value
Age (mean ± SD)	49.41 ± 17.6 years
Female gender [*n*] (% ± MOE)	99/198 (50.0% ± 3.6)
Migration background [*n*] (% ± MOE)	22/196 (11.2% ± 2.3)
Highest level of education [*n*] (% ± MOE)	
• No degree	8 (4.1% ± 1.4)
• Graduated from a general secondary school	26 (13.4% ± 2.4)
• Graduated from intermediate secondary school	51 (26.3% ± 3.2)
• Higher school certificate qualifying for the University of Applied Sciences	30 (15.5% ± 2.6)
• Higher school certificate qualifying for university admission	32 (16.5% ± 2.7)
• University degree	47 (24.2% ± 3.1)
Employment status [*n*] (% ± MOE)	
• Working full time	69 (36.1% ± 3.5)
• Working part-time	29 (15.2% ± 2.6)
• In training/studying	22 (11.5% ± 2.3)
• Job seeking	4 (2.1% ± 1.0)
• Unemployed	4 (2.1% ± 1.0)
• Unable to work	14 (7.3% ± 1.9)
• In retirement	49 (25.7% ± 3.2)
Category of disease [*n*] (% ± MOE)	
• Neurological disease	61 (32.6% ± 3.4%)
• Tumour disease	59 (31.6% ± 3.4%)
• Psychiatric disease	22 (11.8% ± 2.4%)
• Others (i.e. internal disease, orthopaedic disease, etc.)	45 (24.0% ± 3.1%)

*SD* standard deviation, *MOE* margin of error

The results of our questionnaire can be found in Table 2. In summary, 26.6% of respondents rated their knowledge of AI as very good or good, 34.4% rated their knowledge as moderate, 24.1% indicated poor knowledge, and 14.9% indicated no knowledge of AI at all. Most respondents (61.7%) heard about AI in their personal lives but had no direct experience with it. The majority (60.9%) supported the use of AI in healthcare, 36.0% chose the option “I don’t know”, and 3.0% rejected the use of AI. Most respondents indicated that they thought that doctors and AI make the same number of errors (58.4%), 27.3% indicated that doctors make more errors, and 14.3% indicated that AI makes more mistakes. Most respondents (93.2%) indicated that they would be more open to the topic of AI if the doctor explained the AI and its functioning beforehand. When asked about their personal expectations regarding the use of AI in healthcare, 94.5% expected benefits, while 5.5% expected drawbacks. Approval with the use of AI was associated with higher education (*p* = 0.003), personal experience with AI (*p* < 0.001), and if the mechanism of AI was explained (*p* < 0.001).

**Table 2:** Questionnaire items

Item	Value
How would you rate your knowledge of AI in medicine? [*n*] (% ± MOE)	
• No knowledge of AI	29 (14.9 ± 2.5)
• Bad	47 (24.1 ± 3.1)
• Moderate	67 (34.4 ± 3.4)
• Good	42 (21.5 ± 2.9)
• Very good	10 (5.1 ± 1.6)
Have you already had experience with an AI application in your personal life? [*n*] (% ± MOE)	
• I have had my own experience with AI applications.	50 (25.5 ± 3.1)
• I have heard of AI applications but have not had any experience of my own.	121 (61.7 ± 3.5)
• I have never heard of the term AI or had anything to do with it.	25 (12.8 ± 2.4)
How do you feel about the use of AI in medicine? [*n*] (% ± MOE)	
• I am generally against the use of AI.	6 (3.0 ± 1.2)
• I am in favour of the use of AI.	120 (60.9 ± 3.5)
• I don’t know.	71 (36.0 ± 3.4)
What approach would you like to take with regard to your diagnosis? [*n*] (% ± MOE)	
• The physician always makes the diagnosis independently of the result of the AI.	36 (18.8 ± 2.8)
• If the physician is unsure, he/she include the result of the AI in his/her diagnosis.	92 (48.2 ± 3.6)
• The physician always includes AI in their diagnosis.	63 (33.0 ± 3.4)
Who do you think makes more mistakes in diagnostics? [*n*] (% ± MOE)	
• A physician makes more mistakes.	44 (27.3 ± 3.5)
• AI makes more mistakes.	23 (14.3 ± 2.8)
• AI and a physician make the same number of mistakes.	94 (58.4 ± 3.9)
I would be open to the topic of AI if my doctor explained AI and how it works to me beforehand. [*n*] (% ± MOE)	
• Disagree	11 (5.8 ± 1.7)
• Agree less	2 (1.0 ± 0.7)
• Agree partially	35 (18.3 ± 2.8)
• Agree mostly	64 (33.5 ± 3.4)
• Agree fully	79 (41.4 ± 3.6)
What do you personally expect from the use of AI in medicine? [*n*] (% ± MOE)	
• I expect advantages.	172 (94.5 ± 1.7)
• I expect disadvantages.	10 (5.5 ± 1.7)

*MOE* margin of error

### Opinion on the use of AI in specific areas

**Fig. 1:**
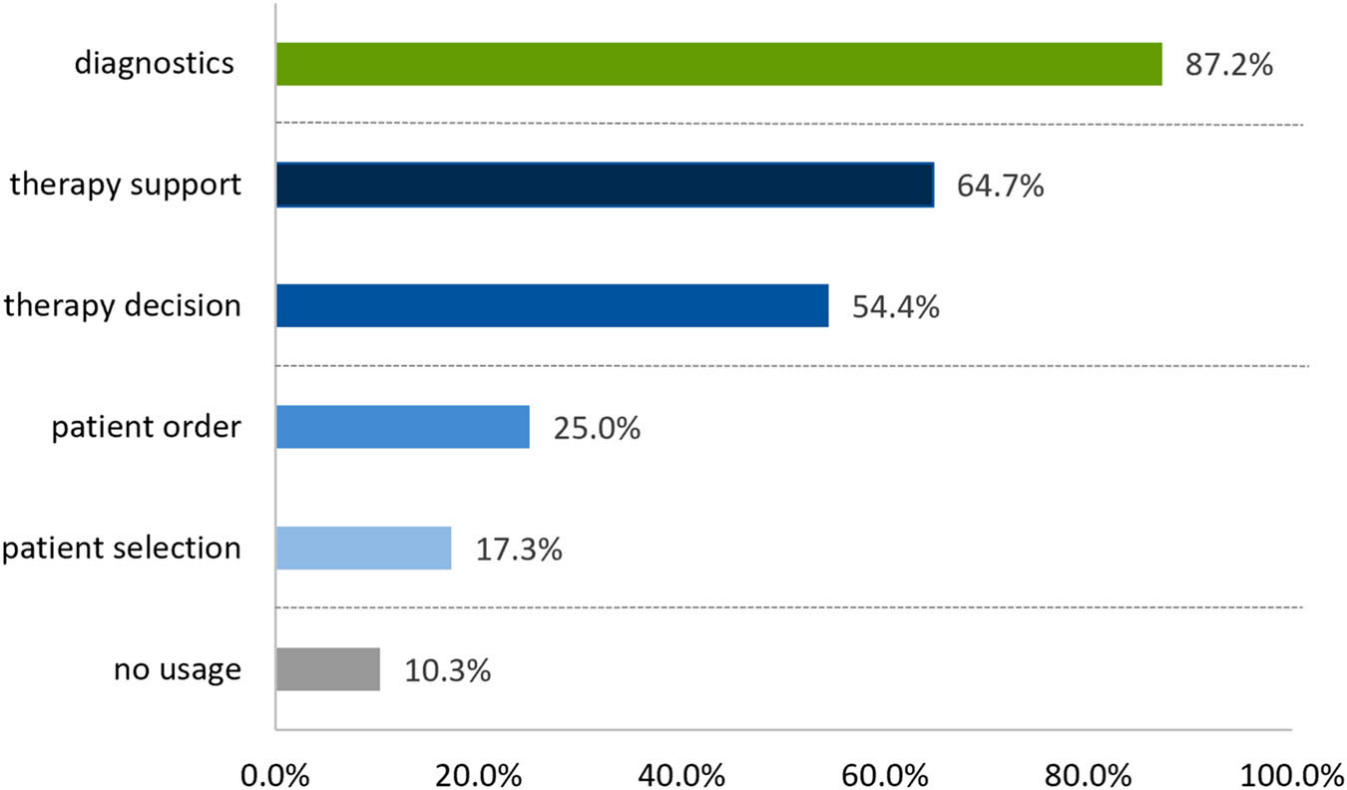
Bar chart showing the frequencies of participants’ answers on the multiple-answer item “I am in favour of the use of artificial intelligence in the following areas”. The use of AI in healthcare is primarily favoured in the areas of diagnostics and therapy (therapy support and therapy decision). There is a comparatively low level of support for the use of AI for triage topics (patient order and patient selection). One tenth of respondents do not want AI to be implemented in any of these areas

The majority supported the use of AI in the fields of diagnostics (87.2% ± 2.7%) and therapy (73.1% ± 3.6%), while only approximately one quarter approved its use for triage (28.2% ± 3.6%). More specifically, 64.7% ± 3.8% approved AI for the support during procedures, whereas the opinion on treatment decision-making was divided (54.5% ± 4.0%). Comparatively fewer participants favoured the use of AI for the order of patients (25.0% ± 3.5%) and for the selection of patients (17.3% ± 3.0%). Overall, 10.3% ± 2.4% of the participants did not want AI to be used in any of these areas. An overview is provided in Fig. 1.

The majority wished to be informed when AI was used in diagnostics (80.8% ± 3.2), treatment decision making (75.0% ± 3.5), and support during procedures (64.1% ± 3.5). Approximately half of patients wished to be informed if AI was used for patient selection (46.2% ± 4.0) and the order of patients (44.9% ± 4.0). Overall, 42.3% ± 4.0 wanted to be informed in all mentioned areas, whereas 16.0% ± 2.9 of participants did not want any information about the use of AI.

In univariate analyses, the use of AI in the fields of diagnostics and therapy was approved by patients who generally approved AI (*p* < 0.001 and *p* < 0.001, respectively), patients with higher knowledge of AI (*p* = 0.017 and *p* = 0.001, respectively), and patients who expected benefits from AI (*p* = 0.005 and *p* = 0.022, respectively). Most patients who generally approved AI (72.6%; 69/95; *p* = 0.723) and patients who expected benefits from AI (69.3%; 95/137; *p* = 0.593) refused its use for triage. However, patients with higher knowledge of AI approved its use for triage (*p* = 0.003).

In multivariable regression analyses, none of the tested variables was independently associated with approval for AI in diagnostics or triage. General acceptance of AI, however, was independently associated with the approval in the field of therapy (OR, 3.0; CI, 1.1–8.0; *p* = 0.032). The model for diagnostics yielded χ² (10) = 15.72, *p* = 0.108, with a Nagelkerke’s *R*^2^ of 0.280. The model for therapy yielded χ² (10) = 16.52, *p* = 0.086, Nagelkerke’s *R*^2^ = 0.167. The model for triage yielded χ² (10) = 10.97, *p* = 0.360, with a Nagelkerke’s *R*^2^ of 0.106. There was no evidence of severe multicollinearity among the predictors.

## Discussion

With our concise questionnaire, we aimed to include a broad spectrum of patients from the ages of 18–87 and with a variety of disorders, ranging from orthopaedic to oncologic conditions. Our findings indicate that patients generally accept and support the use of AI in healthcare, specifically in diagnostics and therapy support. This is in line with the literature [7, 8]. Our major novel finding, however, was the rejection of the use of AI when it comes to patient triage, with more than three-quarters of patients refusing the use of AI in this field. This broad spectrum of positions, with approval and rejection of the use of AI by one and the same patient, depending on the area of application, can explain why very different results are obtained in surveys, especially if no precise areas of AI application are explicitly asked about [9].

A possible explanation for the rejection of the use of AI in the field of triage may be that the subject of “triage” is a topic associated with fear and uncertainty. While a study conducted by Kureda et al showed that the level of knowledge about triage and its benefits is quite high [21], a study conducted by Seibert et al showed that approximately one-third of respondents “did not know why some patients were seen sooner (by the doctor) than others, despite arriving later” [22]. It is conceivable that uncertainty towards the mechanisms of triage, combined with uncertainty towards AI, amplifies disapproval in this field. According to Cadario et al, resistance to the use of medical AI may be caused by difficulty in understanding algorithms (algorithm aversion), while people simultaneously hold on to an illusory understanding of human decision-making in healthcare. Hence, AI is often perceived as a “black box” and therefore rejected [23]. Supporting the hypothesis that a combination of both mechanisms has an impact, 72.6% of patients who supported the general use of AI in healthcare in our study rejected its use in triage. Interestingly, patients with a high degree of knowledge of AI tended to support its use for triage. This is also consistent with the findings of Lennartz et al, who found that “prior knowledge of AI was significantly correlated with the acceptance of AI (…), indicating that patients assigning a higher rating to their prior knowledge of AI were generally more accepting of the use of AI for various aspects of medical treatment and diagnosis” [24]. Our results also indicated that the majority of participants approved the idea of AI as a support system for doctors rather than a substitute, reinforcing the findings of several studies [11, 25, 26]. This supports the concept of the “Knowledge Behaviour Gap” [27]. According to this, more knowledge leads to higher acceptance, which in turn leads to a higher intention to use a technology. Further studies could investigate whether this is due to an objectively better knowledge of AI or whether the subjective assessment is sufficient to increase acceptance. In line with this, 93% of respondents indicated that they would be at least open or positive towards AI if it were explained. Accordingly, a majority of 84.0% wanted to be informed about the use of AI when it was used in at least one of the mentioned areas of diagnostics, therapy, and triage, respectively. Approximately half of our respondents wanted to be informed about its use in all the areas mentioned. This illustrates the patients’ need for information and the necessity of providing information, which can take place in the clinical setting, but also in the form of nationwide information campaigns. There exists a current trend of explainable AI (xAI), which “aims to explain the information behind the black-box model of deep learning that reveals how decisions are made” [28, 29]. This emphasises the desire to understand how AIs arrive at their decisions and how they work.

As to which demographic groups are particularly at risk of being left behind and therefore should be addressed, Fritsch et al and Yakar et al found that older patients, women, and persons with lower education and technical affinity have less trust in AI in healthcare [26, 30]. Notably, none of these characteristics correlated independently with attitudes towards AI in our multivariable analysis. One possible reason is that our sample size is not large enough to allow a valid analysis with regard to specific smaller sociodemographic groups—for example, female patients aged 60 and above with a migration background account for only 3 of our patients. Also, it must be mentioned that both, Yakar et al and Fritsch et al did not analyse patients only, with Yakar et al having investigated the general population—and not patients specifically—and Fritsch et al having included patients and their companions, as well as healthcare professionals. Nonetheless, it still makes sense to pay special attention to the mentioned patient group, because it is generally at risk of being marginalised in healthcare [31]. All in all, our results, with approval and disapproval of AI depending on the field of application, emphasise that rather broad efforts must be undertaken to address the concerns of all patients, not only to improve their understanding and thus acceptance of AI in healthcare, but also to protect potentially marginalised groups from possible disadvantages [32]. Accordingly, the European Parliament already published a study on the topic of “Artificial intelligence in healthcare”, which addresses the need to improve the public’s AI knowledge and skills [33].

## Limitations

Our study has the typical strengths and weaknesses of an exploratory study. Although the size of the study population was adequate for our exploratory approach, our single-site design and the relatively small sample size relative to the number of predictors limit the generalizability of our findings. Nonetheless, they can serve as a foundation for subsequent studies: Future research, ideally involving multiple sites, is warranted to confirm whether our results are applicable across different healthcare environments. While we found no significant association between the type of disease and the responses to the questionnaire, future studies with larger and more diverse samples could explore this in greater depth. A more detailed subgroup analysis would be extremely interesting. From a statistical point of view, however, this makes little sense, as our study is relatively large with a total of around 200 participants, but is still considered exploratory: a subgroup analysis would, for example, lead to a comparison of groups of very varying sizes or groups of 4 vs 5 people, which would not be statistically sound. Nonetheless, our work provides important insights into ensuring that future studies plan their case numbers so that the respective patient subgroups are adequately represented. Future research might benefit from more detailed and mixed-method approaches to capture nuanced perspectives and explore the attitudes of patients towards AI in other clinical fields, countries, and socioeconomic circumstances. It should also be kept in mind that developments in AI are currently progressing so rapidly that our study is only a momentary picture, and patients’ views may well have changed in a short time.

## Conclusion

By using a simple on-site questionnaire, we were able to analyse the views of a broad spectrum of patients on AI in healthcare. Our results show that patients have different opinions about the use of AI in different areas. This should be taken into account when dealing with AI in healthcare. Consistent with the literature, our results imply that patients accept the use of AI in healthcare, specifically in diagnostics and therapy support. However, our novel finding is the rejection of the use of AI for triage.

## Supplementary information

ELECTRONIC SUPPLEMENTARY MATERIAL

## Data Availability

All questionnaires with informed consents and data sets are stored at RWTH Aachen University Hospital.

## Abbreviations

AI: Artificial intelligence

## Declarations

### Guarantor

Omid Nikoubashman.
