# Supplementary material for: Patients’ views on the use of artificial intelligence in healthcare: Artificial Intelligence Survey Aachen (AISA)—a prospective survey
Source: Insights Imaging. 2026 Jan 5;17:6. doi: 10.1186/s13244-025-02159-3 (PMC12770056; doi:10.1186/s13244-025-02159-3)
Supplement: Supplementary file 1 — ELECTRONIC SUPPLEMENTARY MATERIAL [file 13244_2025_2159_MOESM1_ESM.pdf]

Patients’ views on the use of artificial intelligence in healthcare: Artificial Intelligence Survey Aachen (AISA) - a prospective survey

ELECTRONIC SUPPLEMENTARY MATERIAL

Questionnaire on patients' attitudes towards artificial intelligence in medicine

You may have heard that more and more programs and algorithms based on artificial intelligence (AI) have been used in recent years for the early detection of diseases or to support diagnoses.

But what does this mean for the individual patient? What do you personally think about the use of such AI procedures in diagnostics and treatment in medicine?

We would like to hear your opinion as a patient on this topic and have developed the following questionnaire as part of a scientific study. It only takes 5 minutes to complete. No personal data will be passed on to third persons.

Please read all questions carefully and, if possible, do not skip any questions. Only fully completed questionnaires will provide us with findings that we can use for our research and to improve medical care. By taking part, you are making an important contribution to ensuring that AI can be used even more effectively and accepted by patients in the future.

We would like to thank you for your participation and will be happy to answer any questions you may have.

**sample number: AISA -**	**Staff, date:**

Age: __________

Highest educational qualification:

☐ “Hauptschule / Volksschule” ☐ “Realschule / mittlere Reife”
☐ “Fachabitur”
☐ “Abitur“
☐ university degree ☐ no degree

Gender:

☐ male
☐ female ☐ diverse

Employment status:

☐ working full time
☐ working part-time
☐ in training / studying
☐ Job seeker
☐ unemployed
☐ unable to work
☐ in retirement

Country of birth:

____

Nationality:

____

Migratory background:

☐ yes ☐ no

1. **Due to which injury or illness are you currently in our clinic?**

____

2. **How would you rate your knowledge of artificial intelligence in medicine?**

☐ Very good
☐ Good
☐ Moderate
☐ Bad
☐ No knowledge of artificial intelligence

3. **Have you already had experience with an AI application in your personal life?**
☐ I have had my own experience with AI applications.
☐ I have heard of AI applications but have not had any experience of my own.
☐ I have never heard of the term AI or had anything to do with it.

4. **How do you feel about the use of AI in medicine?**
☐ I am generally against the use of AI.
☐ I am in favour of the use of AI. ☐
I don't know.

5. **What approach would you like to take with regard to your diagnosis?** ☐ The physician always makes the diagnosis independently of the result of the artificial intelligence.
☐ If the physician is unsure, he/she includes the result of the artificial intelligence in his/her diagnosis.
☐ The physician always includes the artificial intelligence in their diagnosis.

6. **Who do you think makes more mistakes in diagnostics?** ☐ A physician makes more mistakes.
☐ Artificial intelligence makes more mistakes.
☐ Artificial intelligence and a physician make the same number of mistakes.

For the following statements, please always tick the answer option that applies to you.

7. **I would be open to the topic of artificial intelligence if my doctor explained artificial intelligence and how it works to me beforehand.** ☐ Fully agree. ☐ Mostly agre

   ☐ Partially agree

   ☐ Less agree

   ☐ Disagree

8. **What do you personally expect from the use of AI in medicine?**

   ☐ I expect advantages.

   ☐ I expect disadvantages.

9. **Tick the box.**
   I am in favour of the use of AI in:
   (No cross and multiple crosses possible)

   ☐ **Diagnostics** *(e.g. support in recognising images in radiological images)*

   ☐ **Patient selection** *(e.g. decision on treatment vs. non-treatment in emergencies)*

   ☐ **Patient order** *(e.g. deciding on the order of patients)*

   ☐ **Treatment decision** *(e.g. decision on drug treatment or surgery for cancer)*

   ☐ **Therapy support** *(e.g. to support the selection of materials for an operation)*

10. Tick the box.

I would like to be informed when AI is used for:

(No cross and multiple crosses possible)

☐ **Diagnostics** *(e.g. support in recognising images in radiological images)* ☐ **Patient selection** *(e.g. decision on treatment vs. non-treatment in emergencies)*

☐ **Patient order** *(e.g. deciding on the order of patients)*

☐ **Treatment decision** *(e.g. decision on drug treatment or surgery for cancer)*

☐ **Therapy support** *(e.g. to support the selection of materials for an operation)*

Fragebogen zur Einstellung von Patienten gegenüber Künstlicher Intelligenz in der Medizin

Vielleicht haben Sie schon davon gehört, dass in den letzten Jahren immer mehr Programme und Algorithmen zur Früherkennung von Krankheiten oder zur Unterstützung von Diagnosen eingesetzt werden. Auch in unserer Klinik kommen solche Verfahren bereits zum Einsatz. Diese Algorithmen, auch Künstliche Intelligenz (KI) genannt, stellen zum Beispiel Bilder der Lunge direkt bei der Ausgabe so dar, dass den zuständigen Radiologinnen und Radiologen bestimmte Befunde einfacher auffallen. Dazu wird der KI „beigebracht", wie eine gesunde Lunge aussieht und wie sich bekannte Erkrankungen zeigen.

Doch was bedeutet das für den individuellen Patienten? Was denken Sie persönlich über den Einsatz von solchen KI-Verfahren in der Diagnostik und Behandlung in der Medizin?

Wir möchten gerne Ihre Meinung als Patient zu diesem Thema hören und haben im Rahmen einer wissenschaftlichen Studie den folgenden Fragebogen entwickelt. Die Beantwortung dauert nur 5 Minuten. Es werden keine persönlichen Daten an Dritte weitergegeben.

Bitte lesen Sie alle Fragen sorgfältig durch und lassen Sie nach Möglichkeit keine Fragen aus. Nur vollständig ausgefüllte Bögen liefern uns später Erkenntnisse, die wir für unsere Forschung und zur Verbesserung der medizinischen Versorgung nutzen können. Mit der Teilnahme leisten Sie einen wichtigen Beitrag dazu, dass KI in Zukunft noch besser eingesetzt und von Patienten akzeptiert werden kann.

Wir bedanken uns für Ihre Teilnahme und stehen für Rückfragen gerne zur Verfügung.

Aus Gründen der besseren Lesbarkeit wird bei Personenbezeichnungen und personenbezogenen Hauptwörtern in diesem Fragebogen die männliche Form verwendet. Nichtsdestoweniger beziehen sich die Angaben auf Angehörige aller Geschlechter.

Probandennummer: AISA - **HZ Personal, Datum:**

Alter: _________

Höchster Bildungsabschluss:

☐ Hauptschule / Volksschule ☐ Realschule / mittlere Reife

☐ Fachabitur

☐ Abitur

☐ Studium

☐ kein Abschluss

Geschlecht:

☐ männlich

☐ weiblich

☐ divers

Beschäftigungsstatus:

☐ arbeitend in Vollzeit

☐ arbeitend in Teilzeit

☐ in Ausbildung / Studium

☐ arbeitssuchend

☐ arbeitslos

☐ arbeitsunfähig

☐ im Ruhestand

Geburtsland:

____

Nationalität:

____

Migrationshintergrund:

☐ ja

☐ nein

1. **Aufgrund welcher Verletzung oder Erkrankung sind Sie aktuell in unserer Klinik?**

2. **Wie schätzen Sie ihr Wissen bezüglich Künstlicher Intelligenz in der Medizin ein?**

   ☐ Sehr gut

   ☐ Gut

   ☐ Mittelmäßig

   ☐ Schlecht

   ☐ Kein Wissen über Künstliche Intelligenz

3. **Haben Sie in ihrem persönlichen Leben bereits Erfahrungen mit einer KIAnwendung gemacht?**

   ☐ Ich habe eigene Erfahrungen mit KI-Anwendung gemacht. ☐ Ich habe von KI-Anwendungen gehört, habe aber keine eigenen Erfahrungen gemacht.

   ☐ Ich habe bis heute noch nie von dem Begriff KI gehört oder etwas damit zu tun gehabt.

4. **Wie stehen Sie zum Einsatz von KI in der Medizin?** ☐ Ich lehne den Einsatz von KI generell ab.

   ☐ Ich befürworte den Einsatz von KI. ☐
   Ich weiß nicht.

5. **Welche Vorgehensweise würden Sie sich wünschen?**

   ☐ Der Arzt trifft Entscheidungen immer unabhängig vom Ergebnis der Künstlichen Intelligenz.

   ☐ Wenn der Arzt unsicher ist, bezieht er das Ergebnis der Künstlichen Intelligenz in seine Entscheidung mit ein.

   ☐ Der Arzt bezieht die Künstliche Intelligenz immer in seine Entscheidungen mit ein.

6. **Wer macht Ihrer Meinung nach mehr Fehler in der Diagnostik?** ☐ Ein Arzt macht mehr Fehler.

   ☐ Eine Künstliche Intelligenz macht mehr Fehler.

   ☐ Eine Künstliche Intelligenz und ein Arzt machen gleich viele Fehler.

Bitte kreuzen Sie bei den nachfolgenden Aussagen immer die für Sie zutreffendste Antwortmöglichkeit an.

7. **Ich wäre dem Thema Künstliche Intelligenz offen gegenüber, wenn mein Arzt mir die Künstliche Intelligenz und ihre Funktionsweise vorher erklären würde.**

   ☐ Stimme voll und ganz zu

   ☐ Stimme überwiegend zu

☐ Stimme teilweise zu

☐ Stimme weniger zu

☐ Stimme nicht zu

8. **Was erwarten Sie persönlich von dem Einsatz von KI in der Medizin?**

   ☐ Ich erwarte Vorteile.

   ☐ Ich erwarte Nachteile.

9. **Kreuzen Sie an:**
   Ich befürworte den Einsatz von KI bei:
   (Kein Kreuz und mehrere Kreuze möglich)

   ☐ **Befunderhebung** *(z.B. Unterstützung beim Erkennen von Krankheiten in radiologischen Aufnahmen)*

   ☐ **Patientenauswahl** *(z.B. Entscheidung über Behandlung vs. Nicht-Behandlung bei Notfällen)*

   ☐ **Patientenreihenfolge** *(z.B. Entscheidung über die Behandlungsreihenfolge von Patienten)*

   ☐ **Therapieentscheidung** *(z.B. Entscheidung über medikamentöse Behandlung oder Operation bei Erkrankungen wie Krebs)*

   ☐ **Therapieunterstützung** *(z.B. zur Unterstützung bei der Materialauswahl während einer Operation)*

10. **Kreuzen Sie an:**
    Ich möchte informiert werden, wenn KI verwendet wird bei:
    (Kein Kreuz und mehrere Kreuze möglich)

    ☐ **Befunderhebung** *(z.B. Unterstützung beim Erkennen von Krankheiten in radiologischen Aufnahmen)*

    ☐ **Patientenauswahl** *(z.B. Entscheidung über Behandlung vs. Nicht-Behandlung bei Notfällen)*

☐ **Patientenreihenfolge** *(z.B. Entscheidung über die Behandlungsreihenfolge von Patienten)*

☐ **Therapieentscheidung** *(z.B. Entscheidung über medikamentöse Behandlung oder Operation bei Erkrankungen wie Krebs)*

☐ **Therapieunterstützung** *(z.B. zur Unterstützung bei der Materialauswahl während einer Operation)*
